# A Case Report of Severe Subcutaneous Emphysema Requiring Tracheostomy Following Robot-Assisted Laparoscopic Pancreatectomy

**DOI:** 10.7759/cureus.99443

**Published:** 2025-12-17

**Authors:** Atsuko Kawai, Sakura Okamoto, Hideaki Note, Jyunya Nakada

**Affiliations:** 1 Dentistry, Meieki Dental Clinic Orthodontic, Nagoya, JPN; 2 Anesthesiology, Aichi Medical University Hospital, Nagakute, JPN; 3 Anesthesiology, Aichi Cancer Center, Nagoya, JPN

**Keywords:** airway management, hypercapnia, laparoscopy, obesity, robotic surgery, subcutaneous emphysema, tracheostomy

## Abstract

Subcutaneous emphysema following robotic surgery is a recognized complication, but progression to severe airway compromise is rare. This report discusses unique risk factors and management challenges in an obese patient, contrasting with typical presentations in underweight individuals. An 81-year-old obese female (BMI 26.5 kg/m²) underwent a prolonged (8.9 hours) robot-assisted distal pancreatectomy. She developed extensive subcutaneous emphysema extending to her neck and face, causing severe pharyngeal edema that precluded extubation. Airway management required continued intubation and a tracheostomy on postoperative day 4. The prolonged emphysema resolved in 17 days. This case underscores the need for a high index of suspicion for severe emphysema in patients with any body habitus during prolonged surgery. It also highlights the paramount importance of a cautious airway strategy, prioritizing safety over early extubation when emphysema involves the neck.

## Introduction

Subcutaneous emphysema is a relatively common complication associated with laparoscopic surgery, and in most cases, it resolves spontaneously [1]. However, in rare instances, it can extend to the neck and face, leading to a critical condition involving upper airway obstruction [1,2]. We report the case of an elderly woman who developed severe subcutaneous emphysema following robot-assisted distal pancreatectomy, making extubation difficult due to the risk of airway obstruction and ultimately requiring a tracheostomy after prolonged mechanical ventilation. We analyze the factors contributing to the severity and prolongation of the subcutaneous emphysema in this case and discuss the perioperative management lessons for similar high-risk patients.

## Case presentation

Patient information

An 81-year-old female was scheduled for robot-assisted distal pancreatectomy for pancreatic cancer. Her past medical history included sick sinus syndrome, atrial fibrillation (AF), transient ischemic attack, hypertension, and hypertrophic cardiomyopathy. Her BMI was 26.5 kg/m² (classified as obesity according to the Japan Society for the Study of Obesity criteria).

Anesthetic and surgical course

Tracheal intubation was performed using a McGrath video laryngoscope with a 7.0 mm internal diameter tube. The patient was placed in the reverse Trendelenburg position for the robotic procedure. Anesthesia was managed with total intravenous anesthesia. A standard pneumoperitoneum pressure of 10 mmHg was maintained. Approximately four hours after initiating pneumoperitoneum, end-tidal CO₂ (EtCO₂) began to rise, reaching a maximum of 51 mmHg. Simultaneously, physical examination by the anesthesiologist revealed progressive subcutaneous emphysema on the anterior chest, characterized by severe crepitus.

The surgical team was immediately alerted. Intraoperative interventions included inspecting the port sites, repositioning the trocars, and placing additional sutures to improve the seal. Despite these measures, the emphysema and hypercapnia continued to progress over the prolonged surgical time of eight hours and 54 minutes, extending to the neck and face. Conversion to open surgery was discussed as an option, but it was decided to proceed with the robotic procedure due to the advanced stage of the dissection.

**Figure 1:**
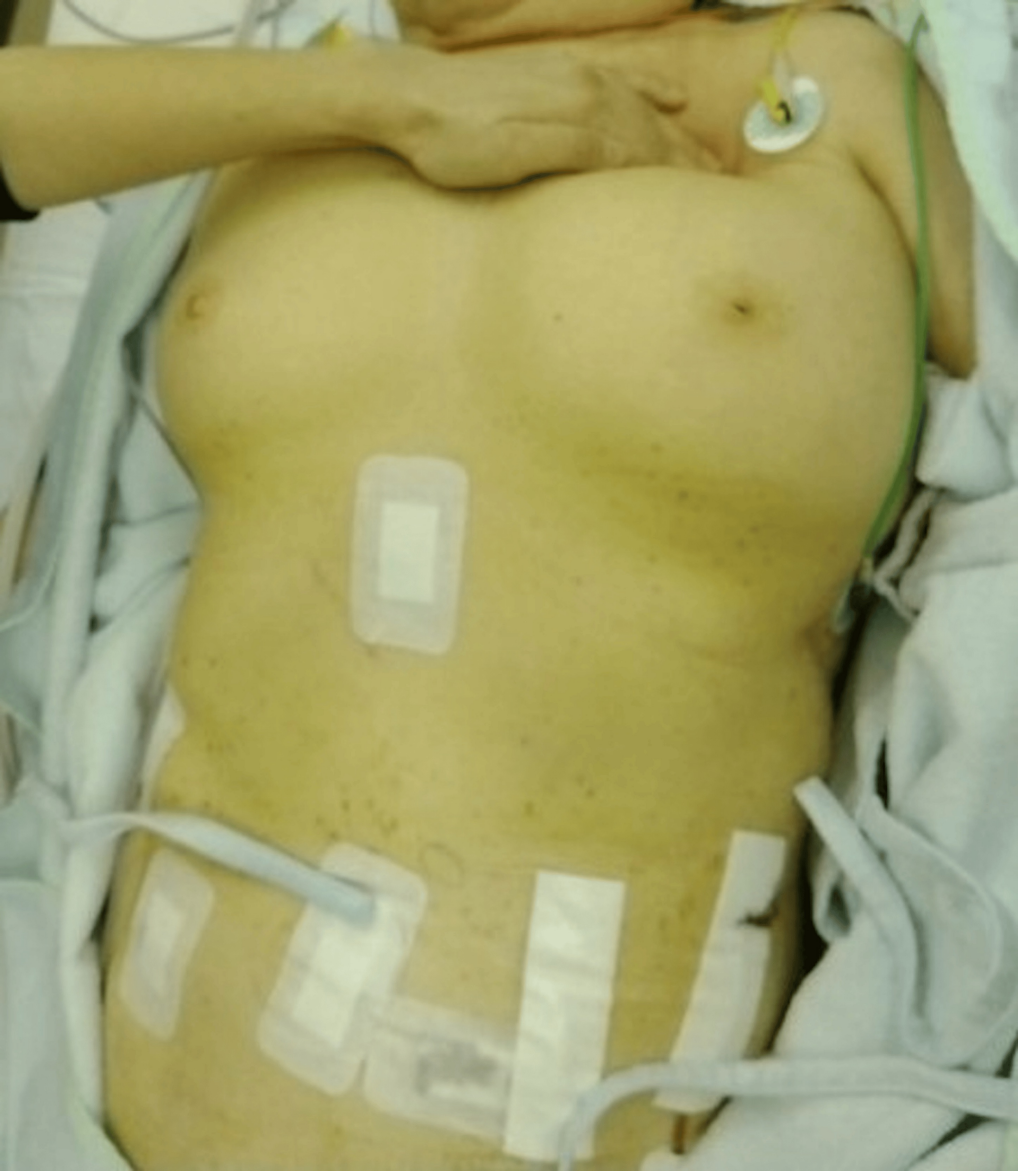
External appearance of the neck and anterior torso upon ICU admission The significant compressibility of the tissue, visualized by the indenting fingers, was associated with severe crepitus on palpation.

At the end of surgery, the patient's appearance was notable for marked swelling from the neck to the anterior chest wall due to subcutaneous emphysema, with obliteration of the clavicular contours and tense, shiny skin (Figure 1).

**Figure 2:**
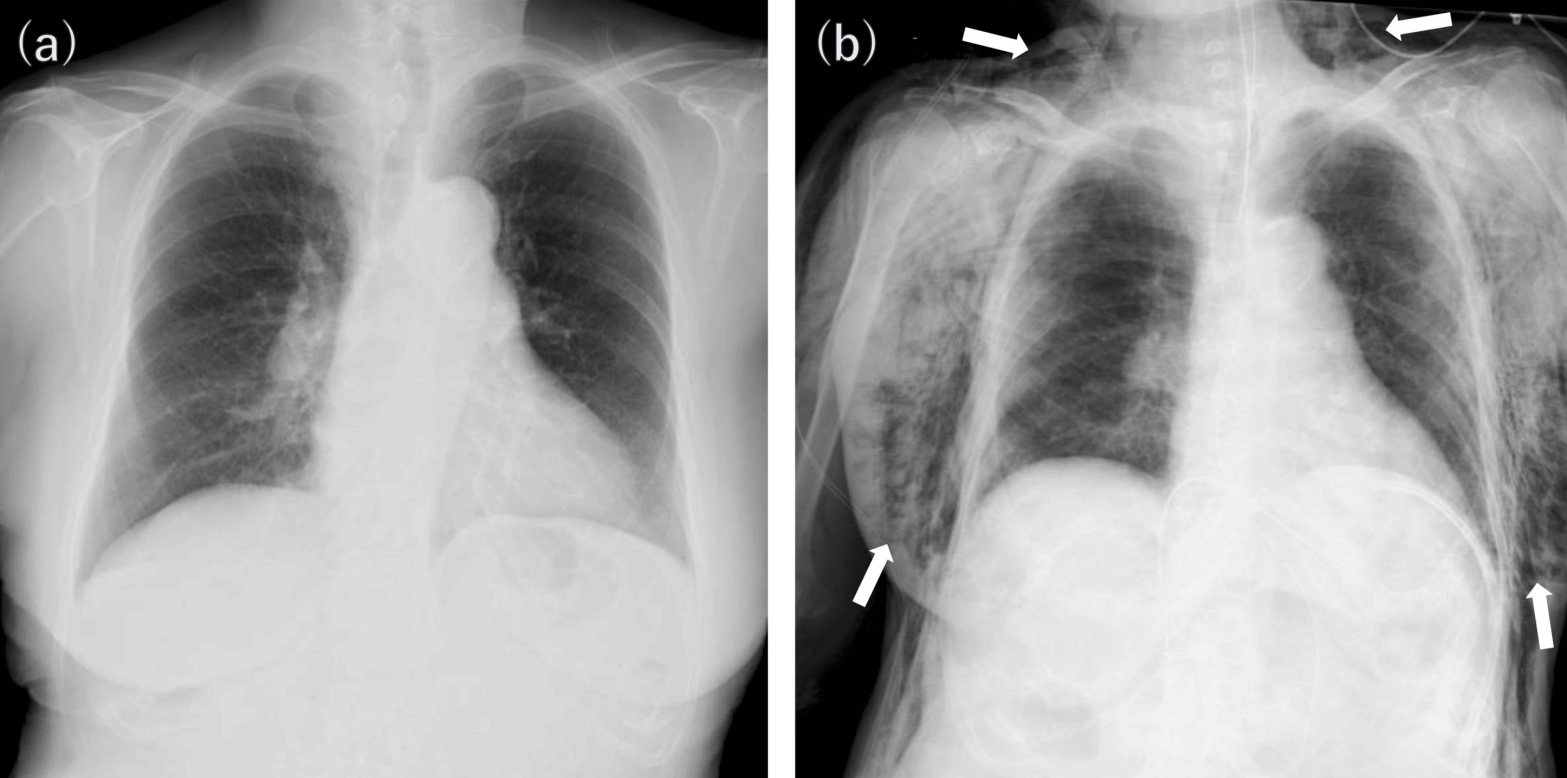
Comparison of preoperative and postoperative chest X-rays (anteroposterior view) (a) The preoperative chest X-ray shows no significant findings. (b) The postoperative X-ray, taken in the operating room, reveals extensive subcutaneous emphysema extending from the neck to the bilateral chest walls, indicated by white arrows. No obvious pneumothorax is identified.

A chest X-ray taken in the operating room revealed extensive subcutaneous emphysema with no associated pneumothorax (Figure 2).

Although the cuff leak test was negative, postoperative fiberoptic examination revealed severe pharyngeal edema. Consequently, the risk of airway obstruction was deemed extremely high, and the patient was transferred to the ICU while remaining intubated.

Postoperative course

**Figure 3:**
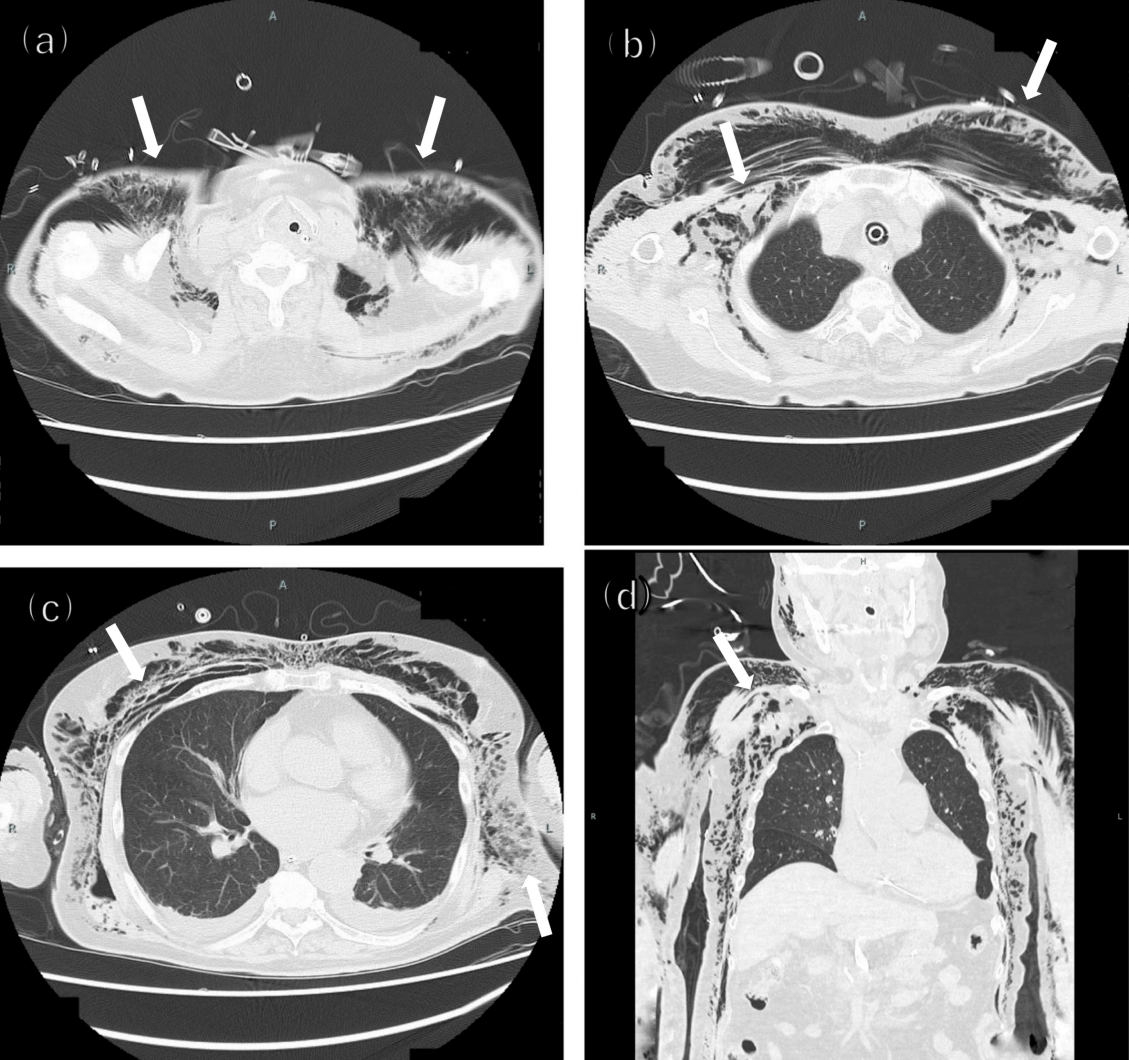
CT images on postoperative day 1, demonstrating severe subcutaneous and intramuscular emphysema (indicated by white arrows) Demonstrating severe subcutaneous and intramuscular emphysema. (a) Axial view at the neck level shows extensive gas surrounding the trachea and within the subcutaneous tissues and sternocleidomastoid muscles. (b) Axial view at the upper thorax level reveals significant emphysema in the subcutaneous and pectoral muscle layers. (c) Axial view at the cardiac level shows continued extensive emphysema throughout the chest wall. (d) The coronal view illustrates the vast craniocaudal extent of the emphysema, stretching from the neck down to the lateral abdominal wall.

The postoperative course was divided into three phases. In Phase 1 (postoperative day (POD) 1-4), the patient was managed with sedation and mechanical ventilation. A chest CT on POD 1 demonstrated extensive subcutaneous and intramuscular emphysema of the neck and chest wall (Figure 3).

The emphysema did not improve, and a tracheostomy was performed on POD 4 to ensure a secure airway. In Phase 2 (POD 5-13), the patient was characterized by complications, including delayed awakening, bleeding from the tracheostomy site following resumption of anticoagulation, and an episode of AF with rapid ventricular response. In Phase 3 (POD 17-40), a CT scan on POD 17 revealed a clear improvement in the emphysema. The patient was gradually weaned from mechanical support and eventually transferred to a rehabilitation facility on POD 40.

## Discussion

Post-laparoscopic subcutaneous emphysema (PLSE) is a known complication, with studies identifying female sex, low BMI, high intraoperative EtCO₂, and prolonged insufflation as risk factors [3,4]. Procedure-related factors, including surgical times exceeding 3.5 hours, improper cannula placement, and inadequate port sealing, have also been emphasized [5]. A large retrospective study found that prolonged operative time (>200 min), elevated EtCO₂ (≥50 mmHg), and the use of multiple ports were independent predictors of subcutaneous emphysema [6]. Furthermore, the use of a robotic approach, as in our patient, may inherently increase this risk, as meta-analyses indicate that robotic surgery is associated with longer operative times than conventional laparoscopy [7]. Our case presented several of these risk factors (female, prolonged surgery, use of multiple ports, and high EtCO₂).

However, a striking feature was the patient's obesity (BMI 26.5 kg/m²), contradicting the commonly cited risk of low BMI. This case raises the hypothesis that "deviation from a standard body habitus" itself could potentially be a risk factor for severe complications. While emaciation may facilitate the initial gas dissection [8], obesity may increase the risk of severe airway compromise once emphysema develops, potentially due to inadequate port sealing in a thick abdominal wall [5].

The persistence of the emphysema until POD 17 was another unique feature. The most plausible explanation is the formation of a closed space with air replacement, where highly absorbable CO₂ was replaced by less soluble nitrogen from surrounding tissues. This hypothesis is clinically relevant, as iatrogenic emphysema caused by air, rather than highly absorbable CO₂, is known to be more persistent and carries a risk of severe complications such as airway compromise [9]. This is in contrast to an air leak from the lungs, which was considered unlikely as no pneumothorax was observed. For such cases, while skin incision is a therapeutic option, securing the airway is of paramount importance. Invasive drainage methods were considered but withheld due to the diffuse nature of the emphysema and the high risk of bleeding associated with necessary anticoagulation for AF. The fact that delayed respiratory distress has been reported post-extubation in a similar case [10] reinforces the validity of our conservative airway management.

A similar case of severe emphysema following robotic surgery has been reported in this journal by Garcia et al., which also required ICU admission. However, their patient was successfully extubated on POD 3 [11]. Our case demonstrates a significantly more severe and prolonged clinical course, ultimately necessitating a tracheostomy due to severe pharyngeal edema and requiring 17 days for emphysema resolution. We speculate that this severe edema was exacerbated by venous congestion resulting from the massive subcutaneous emphysema, which increased tissue pressure in the limited fascial spaces of the neck and impaired venous drainage. This comparison highlights that, while rare, progression to a critical, prolonged airway complication remains a significant risk.

Although PLSE is a recognized complication, it is often considered benign and self-limiting, rarely necessitating surgical intervention or conversion. This perception can lead to a risk of underestimation. However, as this case critically demonstrates, severe and progressive emphysema, particularly in obese patients, can lead to life-threatening airway compromise and a prolonged clinical course. Therefore, when significant progression is noted, anesthesiologists must ensure close communication with the surgical team to promptly re-evaluate the surgical approach, including the possibility of converting to open surgery.

## Conclusions

This case provides two key take-home messages. First, patients with non-standard body habitus undergoing prolonged surgery require a high index of suspicion and vigilant monitoring, reflecting the principle that "attention to detail" is key. Second, when emphysema extends to the neck, a cautious approach prioritizing airway safety over early extubation is crucial and potentially life-saving.
